# TRANSCENDING BOUNDARIES AND BARRIERS: END-OF-LIFE CARE AND QUALITY OF LIFE FOR ASIAN POPULATIONS

**DOI:** 10.1093/geroni/igad104.0724

**Published:** 2023-12-21

**Authors:** Kaipeng Wang, Fei Sun, Iraida Carrion

## Abstract

End-of-life care and quality of life are critical issues for older Asian populations, as they face unique cultural, linguistic, and health-related challenges. This symposium will bring together scholars in this field to discuss the latest research and practices for improving end-of-life care planning and quality of life for older Asian populations. The symposium consists of four papers that focus on different Asian ethnic groups. Zhang and colleagues will use survey data to examine the barriers and sociocultural factors of advance care planning among older Chinese Americans. Xu will present a systematic review on non-medical factors that affect quality of life in the last year among older Chinese. Wang will compare the completion of advance directives and examine its psychosocial factors among older Vietnamese, Filipino, and Indonesian Americans. Using a case study of Somang Society, Lee will discuss how non-profit organizations can navigate social media to provide education and volunteer training to promote advance care planning and healthy aging for older Korean Americans. Collectively, these four papers compare the values, barriers, and resources related to end-of-life care between these groups and propose effective strategies for enhancing end-of-life care planning and quality of life for Asian communities. Findings will inform the development and adaptation of culturally sensitive interventions to promote end-of-life health outcomes for diverse Asian populations. Carrion, the discussant, will address the strengths and limitations of these papers and discuss their contribution to theories, practices, policies, and future research on end-of-life care for minority aging populations.

